# Crystal structure, Hirshfeld surface analysis and inter­action energy and DFT studies of (2*Z*)-4-benzyl-2-(2,4-di­chloro­benzyl­idene)-2*H*-1,4-benzo­thia­zin-3(4*H*)-one

**DOI:** 10.1107/S2056989019013586

**Published:** 2019-10-22

**Authors:** Nada Kheira Sebbar, Brahim Hni, Tuncer Hökelek, Mohamed Labd Taha, Joel T. Mague, Lhoussaine El Ghayati, El Mokhtar Essassi

**Affiliations:** a Laboratoire de Chimie Appliquée et Environnement, Equipe de Chimie Bioorganique Appliquée, Faculté des Sciences, Université Ibn Zohr, Agadir, Morocco; b Laboratoire de Chimie Organique Hétérocyclique URAC 21, Pôle de Compétence Pharmacochimie, Av. Ibn Battouta, BP 1014, Faculté des Sciences, Université Mohammed V, Rabat, Morocco; c Department of Physics, Hacettepe University, 06800 Beytepe, Ankara, Turkey; d Department of Chemistry, Tulane University, New Orleans, LA 70118, USA

**Keywords:** crystal structure, di­hydro­thia­zine, hydrogen bond, π-stacking, Hirshfeld surface

## Abstract

The title compound, C_22_H_15_Cl_2_NOS, contains 1,4-benzo­thia­zine and 2,4-di­­chloro­benzyl­idene units, where the di­hydro­thia­zine ring adopts a screw-boat conformation. In the crystal, inter­molecular C—H_Bnz_⋯O_Thz_ (Bnz = benzene and Thz = thia­zine) hydrogen bonds form corrugated chains extending along the *b*-axis direction which are connected into layers parallel to the *bc* plane by inter­molecular C—H_Methy_⋯S_Thz_ (Methy = methyl­ene) hydrogen bonds, en­closing *R*_4_^4^(22) ring motifs. Offset π-stacking inter­actions between 2,4-di­­chloro­phenyl rings [centroid–centroid = 3.7701 (8) Å] and π-inter­actions which are associated by C—H_Bnz_⋯π(ring) and C—H_Dchlphy_⋯π(ring) (Dchlphy = 2,4-di­chloro­phen­yl) inter­actions may be effective in the stabilization of the crystal structure. The Hirshfeld surface analysis of the crystal structure indicates that the most important contributions for the crystal packing are from H⋯H (29.1%), H⋯C/C⋯H (27.5%), H⋯Cl/Cl⋯H (20.6%) and O⋯H/H⋯O (7.0%) inter­actions. Hydrogen-bonding and van der Waals inter­actions are the dominant inter­actions in the crystal packing. Computational chemistry indicates that in the crystal, the C—H_Bnz_⋯O_Thz_ and C—H_Methy_⋯S_Thz_ hydrogen-bond energies are 55.0 and 27.1 kJ mol^−1^, respectively. Density functional theory (DFT) optimized structures at the B3LYP/6-311G(d,p) level are compared with the experimentally determined mol­ecular structure in the solid state. The HOMO–LUMO behaviour was elucidated to determine the energy gap.

## Abstract 2

The title compound contains 1,4-benzo­thia­zine and 2,4-di­chloro­benzyl­idene units, where the di­hydro­thia­zine ring adopts a screw-boat conformation. In the crystal, inter­molecular C—H_Bnz_⋯O_Thz_ (Bnz = benzene and Thz = thia­zine) hydrogen bonds form corrugated chains extending along the *b*-axis direction which are tied into layers parallel to the *bc* plane by inter­molecular C—H_Methy_⋯S_Thz_ (Methy = methyl­ene) hydrogen bonds, enclosing 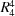
(22) ring motifs.

## Chemical context   

1,4-Benzo­thia­zine derivatives constitute an important class of heterocyclic systems. These mol­ecules exhibit a wide range of biological applications, indicating the fact that the 1,4-benzo­thia­zine moiety is a template potentially useful in medicinal chemistry research and therapeutic applications, such as the anti-inflammatory (Trapani *et al.*, 1985 ▸; Gowda *et al.*, 2011 ▸), anti­pyretic (Warren & Knaus, 1987 ▸), anti­microbial (Armenise *et al.*, 2012 ▸; Rathore & Kumar, 2006 ▸), anti­viral (Malagu *et al.*, 1998 ▸), anti­cancer (Gupta *et al.*, 1985 ▸; Gupta & Gupta, 1991 ▸) and anti-oxidant (Zia-ur-Rehman *et al.*, 2009 ▸) areas. They have also been reported as precursors for the syntheses of new compounds (Sebbar *et al.*, 2015*a* ▸; Vidal *et al.*, 2006 ▸) possessing anti­diabetic (Tawada *et al.*, 1990 ▸) and anti­corrosion activities (Ellouz *et al.*, 2016*a* ▸,*b* ▸; Sebbar *et al.*, 2016*a* ▸). They also possess biological properties (Hni *et al.*, 2019*a* ▸,*b* ▸; Sebbar *et al.*, 2017 ▸; Ellouz *et al.*, 2017*a* ▸,*b* ▸, 2018 ▸). As a continuation of our research on the development of *N*-substituted 1,4-benzo­thia­zine derivatives and the evaluation of their potential pharmacological activities, we report here the synthesis of (2*Z*)-4-benzyl-2-(2,4-di­chloro­benzyl­idene)-2*H*-1,4-benzo­thia­zin-3(4*H*)-one, (I), by the reaction of benzyl chloride with (*Z*)-2-(2,4-di­chloro­benzyl­idene)-2*H*-1,4-benzo­thia­zin-3(4*H*)-one and po­tassium carbonate in the presence of tetra-*n*-butyl­ammonium bromide (as catalyst). The mol­ecular and crystal structures, together with the Hirshfeld surface analysis, the inter­molecular inter­action energies and density functional theory (DFT) computational calculations were carried out at the B3LYP/6-311G(d,p) and B3LYP/6-311G(d,p) levels, respectively, for (I) (see Scheme 1).
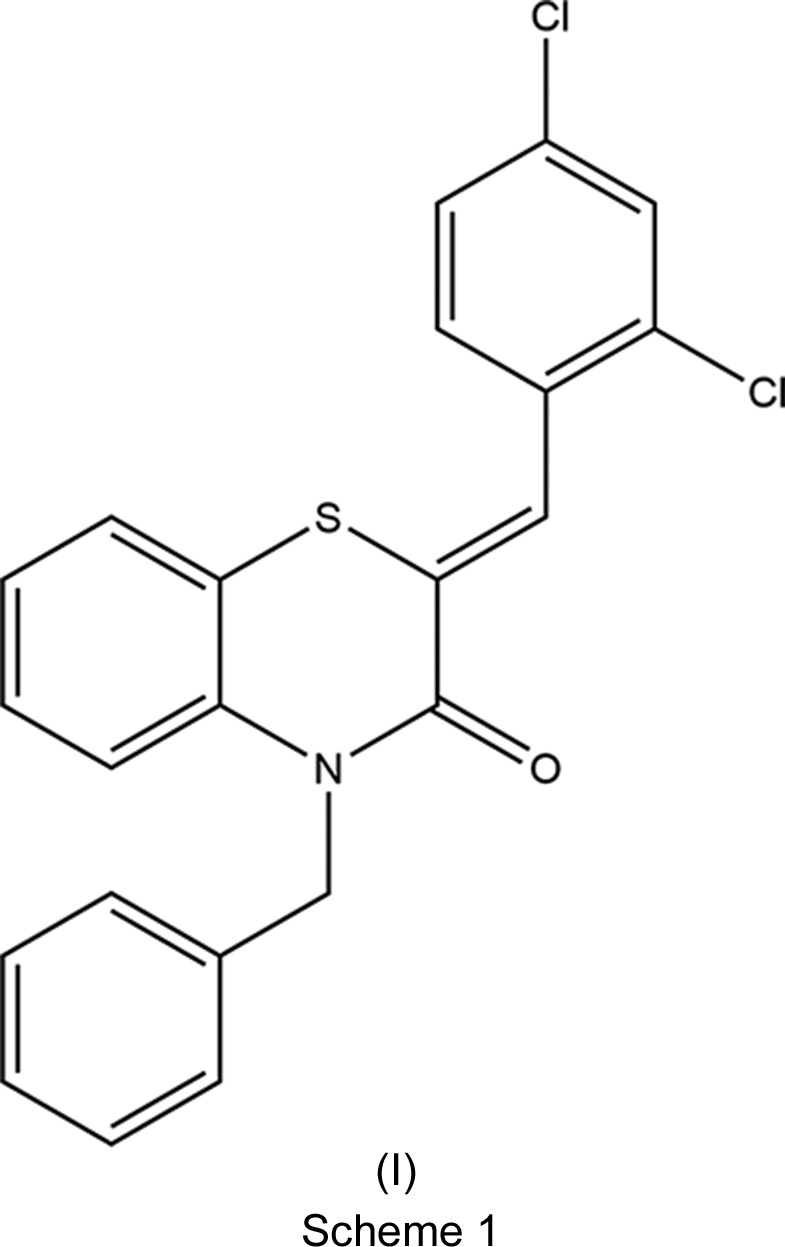


## Structural commentary   

**Figure 1:**
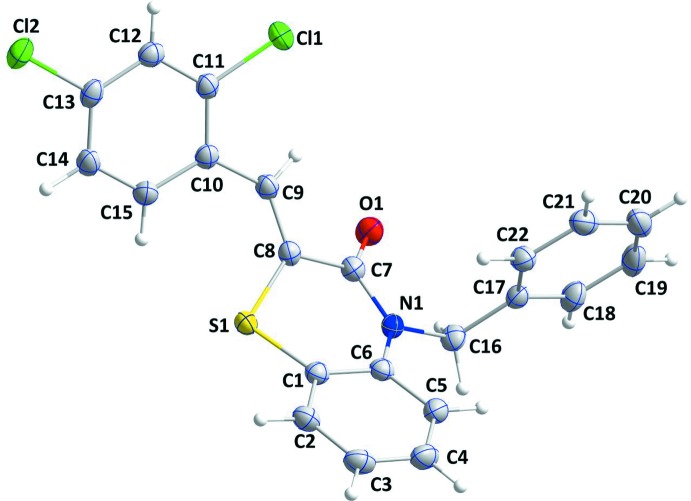
The mol­ecular structure of the title compound with the atom-numbering scheme. Displacement ellipsoids are drawn at the 50% probability level.

The title compound, (I), contains 1,4-benzo­thia­zine and 2,4-di­chloro­benzyl­idene units (Fig. 1 ▸), where the di­hydro­thia­zine ring, *B* (atoms S1/N1/C1/C6–C8), adopts a screw-boat conformation with puckering parameters (Cremer & Pople, 1975 ▸) of *Q*_T_ = 0.4331 (10) Å, θ = 68.34 (16)° and φ = 333.95 (17)°. The planar rings *A* (C1–C6), *C* (C10–C15) and *D* (C17–C22) are oriented at dihedral angles of *A*/*C* = 60.49 (4)°, *A*/*D* = 79.69 (4)° and *C*/*D* = 41.29 (4)°. Atoms Cl1 and Cl2 are −0.0156 (3) and 0.0499 (4) Å from ring *C* and so are almost coplanar.

## Supra­molecular features   

**Figure 2:**
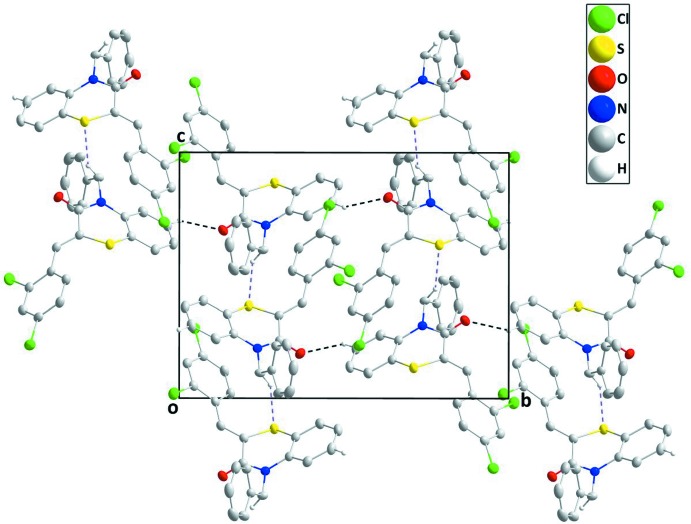
A partial packing diagram, viewed along the *a*-axis direction, with C—H_Bnz_⋯O_Thz_ and C—H_Methy_⋯S_Thz_ (Bnz = benzene, Thz = thia­zine and Methy = methyl­ene) hydrogen bonds shown, respectively, as black and light-purple dashed lines.

In the crystal, inter­molecular C—H_Bnz_⋯O_Thz_ (Bnz = benzene and Thz = thia­zine) hydrogen bonds form corrugated chains extending along the *b*-axis direction which are connected into layers parallel to the *bc* plane by inter­molecular C—H_Methy_⋯S_Thz_ (Methy = methyl­ene) hydrogen bonds, enclosing 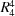
(22) ring motifs (Bernstein *et al.*, 1995 ▸) (Table 1 ▸ and Fig. 2 ▸). Offset π-stacking inter­actions between 2,4-di­chloro­phenyl rings *C* [atoms C10–C15; *Cg*3⋯*Cg*3^i^, where *Cg*3 is the centroid of ring *C*; symmetry code: (i) −*x*, −*y* + 1, −*z* + 1], may further stabilize the structure, with a centroid–centroid distance of 3.7701 (8) Å, together with π-inter­actions, *i.e.* C—H_Bnz_⋯π(ring) and C—H_Dchlphy_⋯π(ring) (Dchlphy = 2,4-di­chloro­phen­yl). The Hirshfeld surface analysis of the crystal structure indicates that the most important contributions for the crystal packing are from H⋯H (29.1%), H⋯C/C⋯H (27.5%), H⋯Cl/Cl⋯H (20.6%) and O⋯H/H⋯O (7.0%) inter­actions. Hydrogen-bonding and van der Waals inter­actions are the dominant inter­actions in the crystal packing.

## Hirshfeld surface analysis   

**Figure 3:**
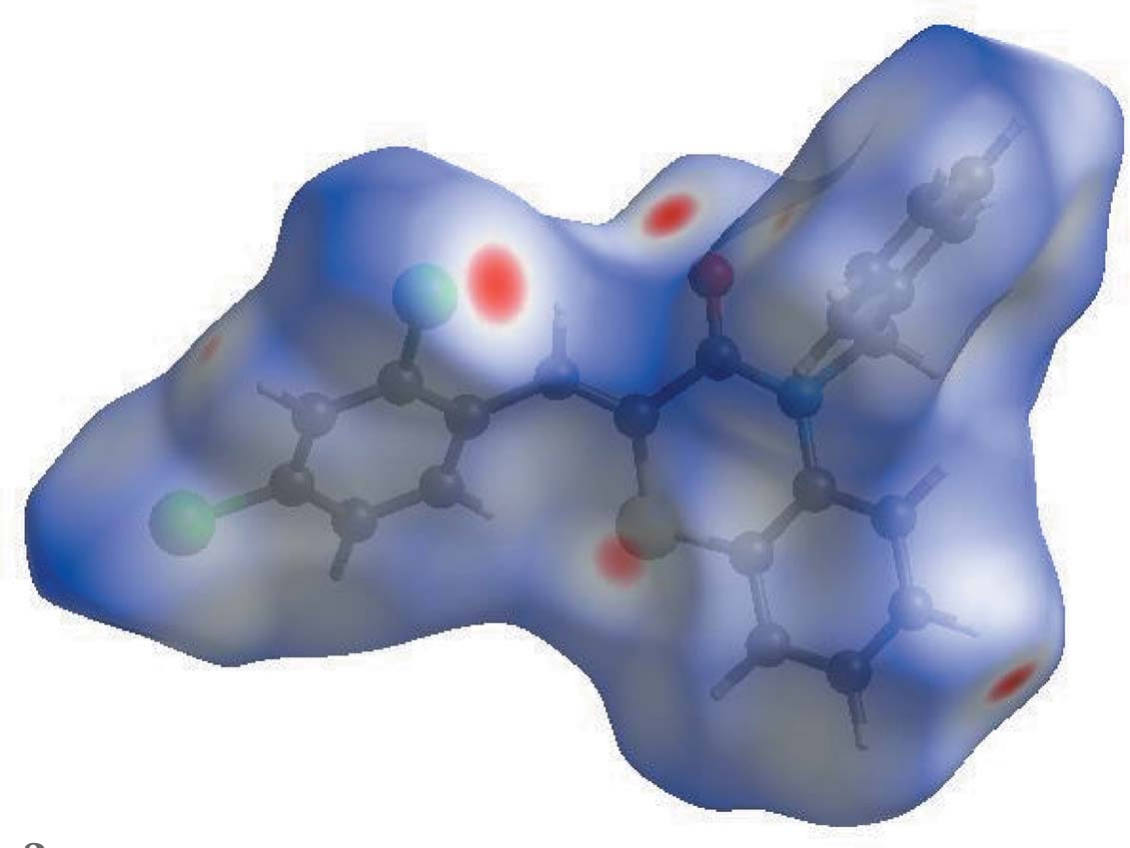
View of the 3D Hirshfeld surface of the title compound, plotted over *d*_norm_ in the range −0.1634 to 1.5051 a.u.

**Figure 4:**
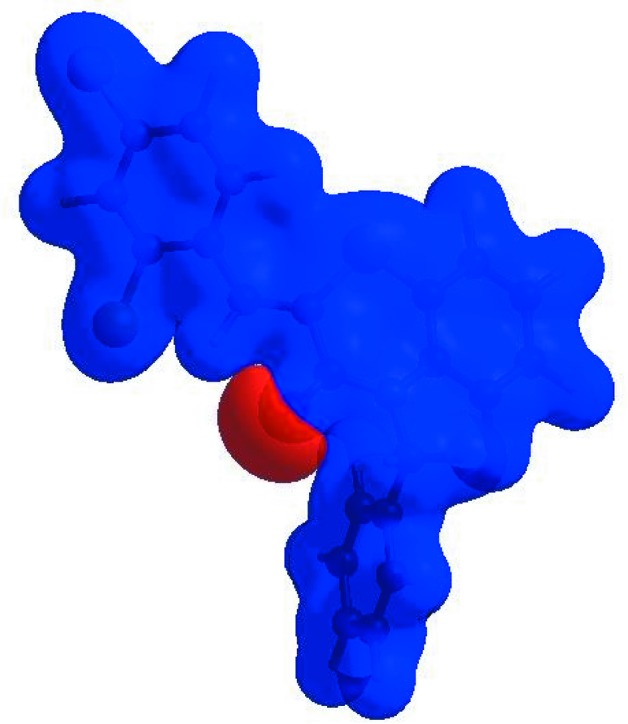
View of the 3D Hirshfeld surface of the title compound, plotted over electrostatic potential energy in the range −0.0500 to 0.0500 a.u., using the STO-3G basis set at the Hartree–Fock level of theory. Hydrogen-bond donors and acceptors are shown as blue and red regions around the atoms corresponding to positive and negative potentials, respectively.

**Figure 5:**
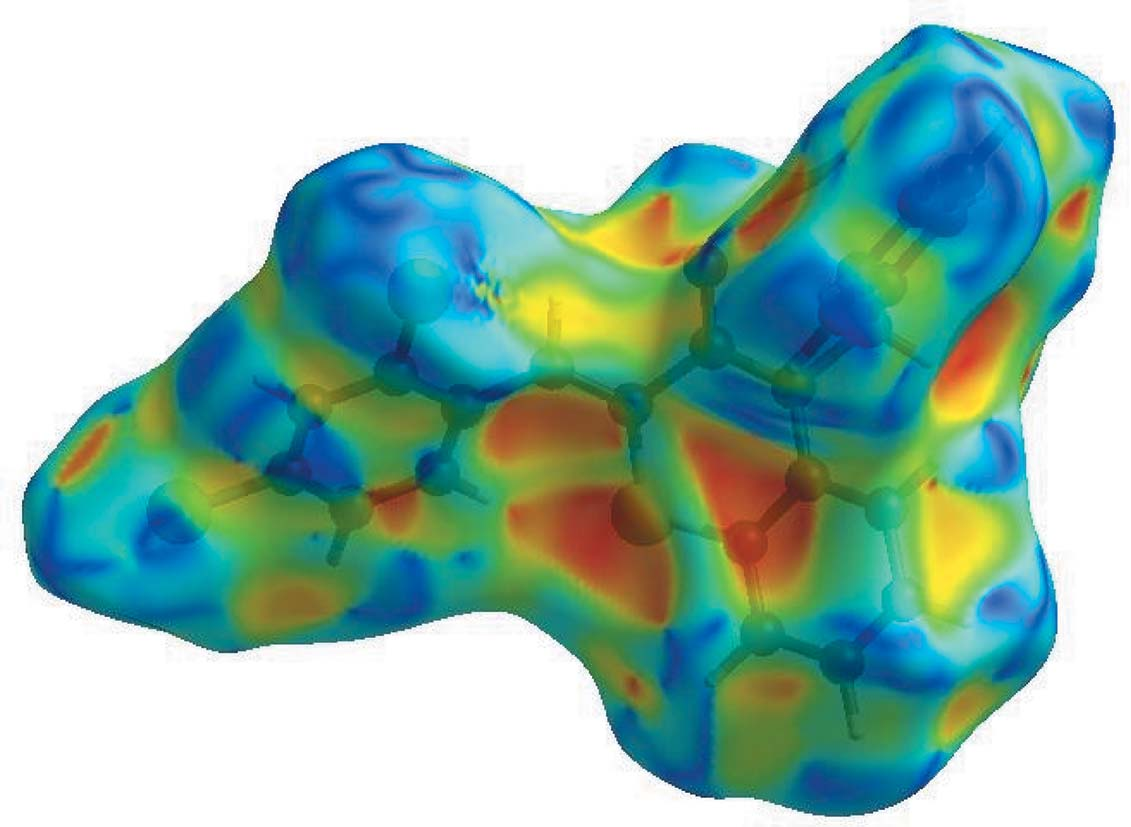
Hirshfeld surface of the title compound plotted over shape-index.

**Figure 6:**
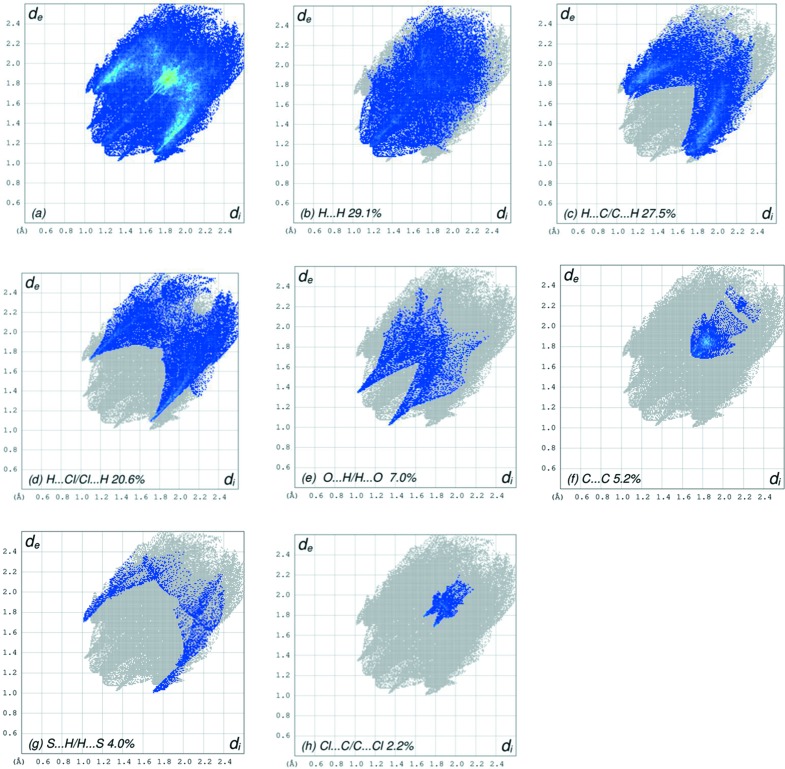
The full 2D fingerprint plots for the title compound, showing (*a*) all inter­actions, and delineated into (*b*) H⋯H, (*c*) H⋯C/C⋯H, (*d*) H⋯Cl/Cl⋯H, (*e*) O⋯H/H⋯O, (*f*) C⋯C, (*g*) S⋯H/H⋯S and (*h*) Cl⋯C/C⋯Cl inter­actions. The *d*_i_ and *d*_e_ values are the closest inter­nal and external distances (in Å) from given points on the Hirshfeld surface contacts.

In order to visualize the inter­molecular inter­actions in the crystal of (I), a Hirshfeld surface (HS) analysis (Hirshfeld, 1977 ▸; Spackman & Jayatilaka, 2009 ▸) was carried out using *CrystalExplorer* (Version 17.5; Turner *et al.*, 2017 ▸). In the HS plotted over *d*_norm_ (Fig. 3 ▸), the white surface indicates contacts with distances equal to the sum of the van der Waals radii, and the red and blue colours indicate distances shorter (in close contact) or longer (distinct contact) than the van der Waals radii, respectively (Venkatesan *et al.*, 2016 ▸). The bright-red spots appearing near atoms O1, S1 and H4 indicate their roles as the respective donors and/or acceptors; they also appear as blue and red regions corresponding to positive and negative potentials on the HS mapped over electrostatic potential (Spackman *et al.*, 2008 ▸; Jayatilaka *et al.*, 2005 ▸), as shown in Fig. 4 ▸. The blue regions indicate the positive electrostatic potential (hydrogen-bond donors), while the red regions indicate the negative electrostatic potential (hydrogen-bond acceptors). The shape-index of the HS is a tool to visualize the π–π stacking by the presence of adjacent red and blue triangles; if there are no adjacent red and/or blue triangles, then there are no π–π inter­actions. Fig. 5 ▸ clearly suggest that there are π–π inter­actions in (I). The overall two-dimensional (2D) fingerprint plot (Fig. 6 ▸ *a*) and those delineated into H⋯H, H⋯C/C⋯H, H⋯Cl/Cl⋯H, O⋯H/H⋯O, C⋯C, S⋯H/H⋯S and Cl⋯C/C⋯Cl contacts (McKinnon *et al.*, 2007 ▸) are illustrated in Figs. 6 ▸(*b*)–(*h*), respectively, together with their relative contributions to the Hirshfeld surface. The most important inter­action is H⋯H, contributing 29.1% to the overall crystal packing, which is reflected in Fig. 6 ▸(*b*) as widely scattered points of high density due to the large hydrogen content of the mol­ecule with the tip at *d*_e_ = *d*_i_ = 1.17 Å, due to the short inter­atomic H⋯H contacts (Table 2 ▸). In the presence of C—H⋯π inter­actions, the pairs of characteristic wings resulting in the fingerprint plot delineated into H⋯C/C⋯H contacts (Fig. 6 ▸ *c*), with a 27.5% contribution to the HS, arises from the H⋯C/C⋯H contacts (Table 2 ▸) and are viewed as pairs of spikes with the tips at *d*_e_ + *d*_i_ = 2.82 and 2.78 Å for thin and thick spikes, respectively. The pair of scattered points of the wings resulting in the fingerprint plots delineated into H⋯Cl/Cl⋯H (Fig. 6 ▸ *d*), with a 20.6% contribution to the HS, has a symmetrical distribution of points with the edges at *d*_e_ + *d*_i_ = 2.78 Å arising from the H⋯Cl/Cl⋯H contacts (Table 2 ▸). The pair of characteristic wings resulting in the fingerprint plot delineated into O⋯H/H⋯O contacts (Fig. 6 ▸ *e*), with a 7.0% contribution to the HS, arises from the O⋯H/H⋯O contacts (Table 2 ▸) and is viewed as a pair of spikes with the tips at *d*_e_ + *d*_i_ = 2.35 Å. The C⋯C contacts (Fig. 6 ▸ *f*) have an arrow-shaped distribution of points with the tip at *d*_e_ = *d*_i_ = 1.7 Å. Finally, the characteristic wings resulting in the fingerprint plots delineated into S⋯H/H⋯S and Cl⋯C/C⋯Cl contacts (Figs. 6 ▸ *g* and 6*h*), with 4.0 and 2.2% contributions to the HS, arise from the S⋯H/H⋯S and Cl⋯C/C⋯Cl contacts (Table 2 ▸) and are viewed with the tips at *d*_e_ = *d*_i_ = 2.70 Å and *d*_e_ + *d*_i_ = 3.46 Å, respectively.

**Figure 7:**
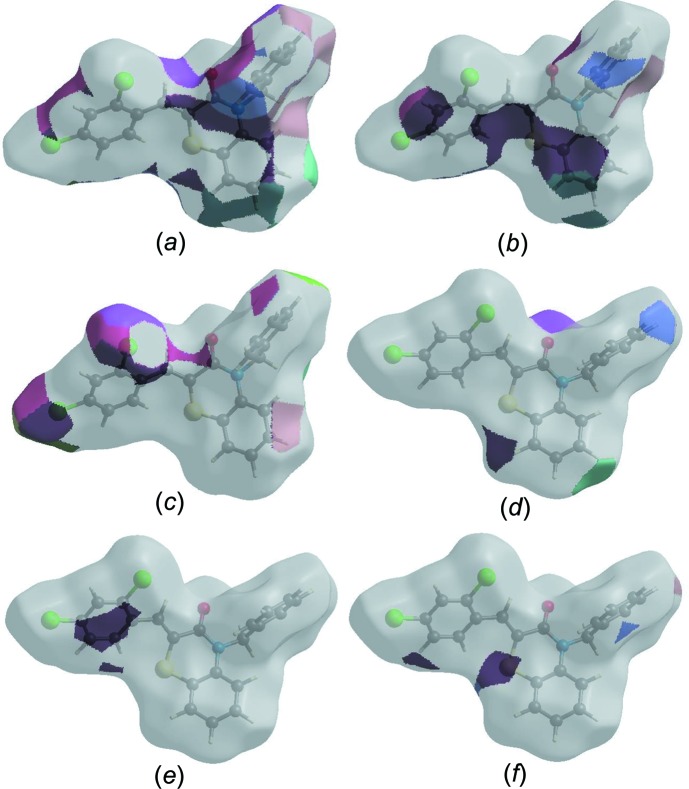
The Hirshfeld surface representations with the function *d*_norm_ plotted onto the surface for (*a*) H⋯H, (*b*) H⋯C/C⋯H, (*c*) H⋯Cl/Cl⋯H, (*d*) O⋯H/H⋯O, (*e*) C⋯C and (*f*) S⋯H/H⋯S inter­actions.

The Hirshfeld surface representations with the function *d*_norm_ plotted onto the surface are shown for the H⋯H, H⋯C/C⋯H, H⋯Cl/Cl⋯H, O⋯H/H⋯O, C⋯C and S⋯H/H⋯S inter­actions in Figs. 7 ▸(*a*)–(*f*), respectively.

The Hirshfeld surface analysis confirms the importance of H-atom contacts in establishing the packing. The large number of H⋯H, H⋯C/C⋯H, H⋯Cl/Cl⋯H and O⋯H/H⋯O inter­actions suggest that van der Waals inter­actions and hydrogen bonding play the biggest roles in the crystal packing (Hathwar *et al.*, 2015 ▸).

## Inter­action energy calculations   

The inter­molecular inter­action energies are calculated using CE–B3LYP/6-31G(d,p) energy model available in *CrystalExplorer* (CE) (Version 17.5; Turner *et al.*, 2017 ▸), where a cluster of mol­ecules would need to be generated by applying crystallographic symmetry operations with respect to a selected central mol­ecule within a default radius of 3.8 Å (Turner *et al.*, 2014 ▸). The total inter­molecular energy (*E*_tot_) is the sum of the electrostatic (*E*_ele_), polarization (*E*_pol_), dispersion (*E*_dis_) and exchange-repulsion (*E*_rep_) energies (Turner *et al.*, 2015 ▸), with scale factors of 1.057, 0.740, 0.871 and 0.618, respectively (Mackenzie *et al.*, 2017 ▸). Hydrogen-bonding inter­action energies (in kJ mol^−1^) were calculated as −20.3 (*E*_ele_), −2.6 (*E*_pol_), −79.4 (*E*_dis_), 60.7 (*E*_rep_) and −55.0 (*E*_tot_) for C—H_Bnz_⋯O_Thz_ hydrogen-bonding inter­actions, and −5.8 (*E*_ele_), −1.0 (*E*_pol_), −51.0 (*E*_dis_), 39.3 (*E*_rep_) and −27.1 (*E*_tot_) for C—H_Methy_⋯S_Thz_ hydrogen-bonding inter­actions.

## DFT calculations   

**Figure 8:**
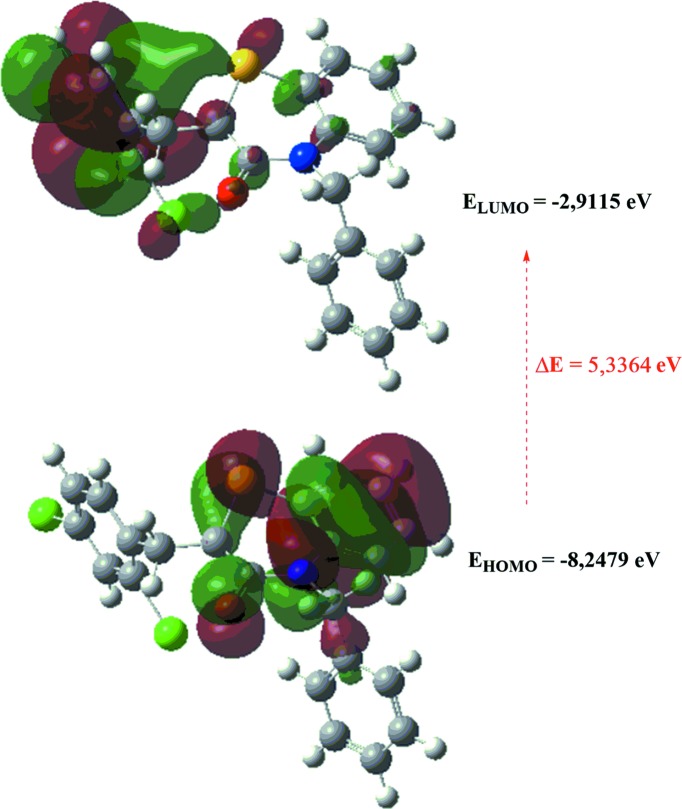
The energy band gap of the title compound.

The optimized structure of (I) in the gas phase was generated theoretically *via* density functional theory (DFT) using standard B3LYP functional and 6-311G(d,p) basis-set calculations (Becke, 1993 ▸), as implemented in *GAUSSIAN09* (Frisch *et al.*, 2009 ▸). The theoretical and experimental results were in good agreement (Table 3 ▸). The highest-occupied mol­ecular orbital (HOMO), acting as an electron donor, and the lowest-unoccupied mol­ecular orbital (LUMO), acting as an electron acceptor, are very important parameters for quantum chemistry. When the energy gap is small, the mol­ecule is highly polarizable and has high chemical reactivity. The DFT calculations provide some important information on the reactivity and site selectivity of the mol­ecular framework. *E*_HOMO_ and *E*_LUMO_ clarifying the inevitable charge exchange collaboration inside the studied material, electronegativity (χ), hardness (η), potential (μ), electrophilicity (ω) and softness (*σ*) are recorded in Table 4 ▸. The significance of η and σ is to evaluate both the reactivity and stability. The electron transition from the HOMO to the LUMO energy level is shown in Fig. 8 ▸. The HOMO and LUMO are localized in the plane extending from the whole mol­ecule. The energy band gap (Δ*E* = *E*_LUMO_ – *E*_HOMO_) of the mol­ecule was about 5.3364 eV, and the frontier mol­ecular orbital (FMO) energies, *E*_HOMO_ and *E*_LUMO_, were −8.2479 and −2.9115 eV, respectively.

## Database survey   

A search in the Cambridge Structural Database (Groom *et al.*, 2016 ▸; updated to June 2019) for compounds containing the fragment II (with *R*_1_ = Ph and *R*_2_ = C; see Scheme 2) gave 14 hits. With *R*_1_ = Ph and *R*_2_ = CH_2_C≡CH (**IIa**) (Sebbar *et al.*, 2014*a* ▸), CH_2_COOH (**IIb**) (Sebbar *et al.*, 2016*c* ▸), 2-(2-oxo-1,3-oxazolidin-3-yl)ethyl (**IIc**) (Sebbar *et al.*, 2016*b* ▸) and (3-phenyl-4,5-dihydro-1,2-oxazol-5-yl)methyl (**IIf**) (Sebbar *et al.*, 2015*b* ▸)] (Scheme 2), there are other examples with *R*_1_ = 4-FC_6_H_4_ and *R*_2_ = CH_2_C≡CH (**IIa**) (Hni *et al.*, 2019*a* ▸), *R*_1_ = 4-ClC_6_H_4_ and *R*_2_ = CH_2_Ph2 (**IId**) (Ellouz *et al.*, 2016*c* ▸), and *R*_1_ = 2-ClC_6_H_4_ and *R*_2_ = CH_2_C≡CH (**IIa**) (Sebbar *et al.*, 2017 ▸) (Scheme 2). In all compounds, the configuration about the benzyl­idene-group C=CHC_6_H_5_ bond is *Z*, and in the majority of these, the heterocyclic ring is quite nonplanar, with the dihedral angle between the plane defined by the benzene ring plus the N and S atoms, and that defined by the N and S atoms and the other two C atoms separating them ranging from *ca* 29 (for **IIa**) to 36° (for **IIf**). The other two (**IIa** and **IIc**) have the benzo­thia­zine unit nearly planar, with corresponding dihedral angles of *ca* 3–4°.
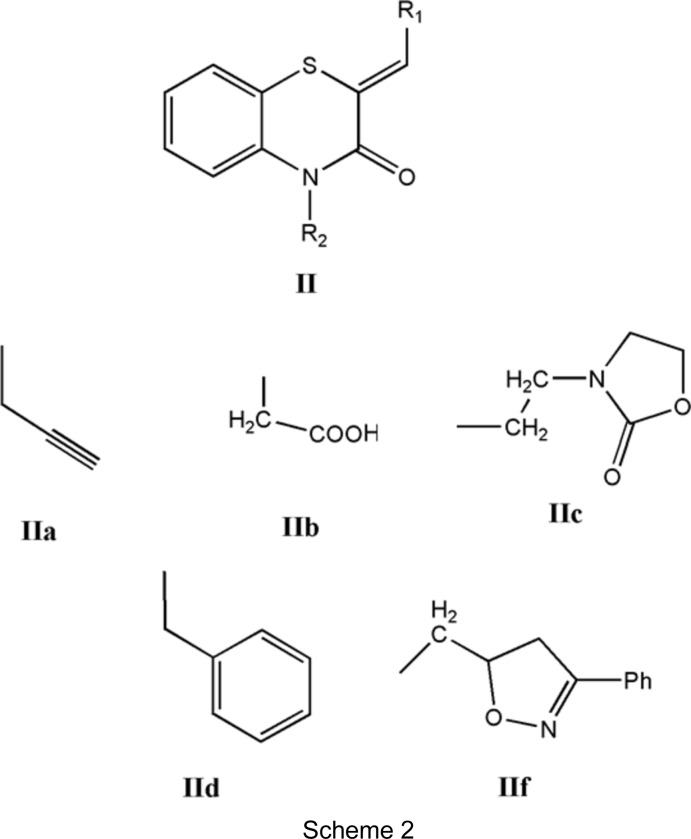


## Synthesis and crystallization   

To a solution of (*Z*)-2-(2,4-di­chloro­benzyl­idene)-2*H*-1,4-benzo­thia­zin-3(4*H*)-one (3.21 mmol), benzyl chloride (6.52 mmol) and potassium carbonate (6.51 mmol) in di­methyl­formamide (DMF; 17 ml) was added a catalytic amount of tetra-*n*-butyl­ammonium bromide (0.33 mmol). The mixture was stirred for 24 h. The solid material was removed by filtration and the solvent evaporated under vacuum. The solid product was purified by recrystallization from ethanol to afford colourless crystals in 82% yield.

## Refinement   

The experimental details, including the crystal data, data collection and refinement, are summarized in Table 5 ▸. H atoms were located in a difference Fourier map and refined freely.

## Supplementary Material

Crystal structure: contains datablock(s) I, global. DOI: 10.1107/S2056989019013586/lh5925sup1.cif

Structure factors: contains datablock(s) I. DOI: 10.1107/S2056989019013586/lh5925Isup2.hkl

Supporting information file. DOI: 10.1107/S2056989019013586/lh5925Isup3.cdx

CCDC references: 1957875, 1957875

Additional supporting information:  crystallographic information; 3D view; checkCIF report

## Figures and Tables

**Table 1:** Hydrogen-bond geometry (Å, °) *Cg*1 and *Cg*4 are the centroids of rings *A* (C1–C6) and *D* (C17–C22), respectively.

*D*—H⋯*A*	*D*—H	H⋯*A*	*D*⋯*A*	*D*—H⋯*A*
C4—H4⋯O1^ix^	0.936 (19)	2.51 (2)	3.3346 (17)	147.7 (15)
C16—H16*B*⋯S1^v^	0.945 (16)	2.852 (16)	3.7011 (13)	149.9 (12)
C3—H3⋯*Cg*4^ix^	0.938 (17)	2.901 (17)	3.6428 (15)	136.8 (13)
C14—H14⋯*Cg*4^x^	0.971 (19)	2.710 (18)	3.5593 (15)	146.8 (14)
C18—H18⋯*Cg*1^xi^	0.979 (18)	2.969 (18)	3.6759 (16)	130.0 (13)

Symmetry codes: (v) 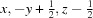; (ix) 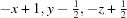; (x) 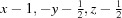; (xi) 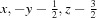.

**Table 2:** Selected interatomic distances (Å)

Cl1⋯Cl1^i^	3.2439 (5)	C6⋯C22	3.4830 (18)
Cl1⋯C14^ii^	3.4981 (14)	C6⋯C12^v^	3.5828 (18)
Cl1⋯H9	2.647 (16)	C7⋯C22	3.4391 (18)
Cl2⋯H19^iii^	2.96 (2)	C10⋯C12^ii^	3.4871 (18)
Cl2⋯H9^ii^	3.044 (16)	C14⋯C20^iv^	3.572 (2)
Cl2⋯H4^iv^	3.138 (18)	C5⋯H16*A*	2.563 (16)
S1⋯Cl2^v^	3.5832 (5)	C6⋯H22	2.904 (15)
S1⋯Cl2^v^	3.5832 (5)	C8⋯H15	2.929 (18)
S1⋯N1	3.0801 (11)	C16⋯H5	2.556 (18)
S1⋯C15	3.1625 (14)	C17⋯H5	2.829 (18)
S1⋯C13^v^	3.6033 (13)	C18⋯H3^vi^	2.998 (17)
S1⋯H15	2.578 (18)	C21⋯H12^i^	2.845 (18)
O1⋯C17	3.2096 (16)	H14⋯C20^iv^	2.964 (18)
O1⋯C4^vi^	3.3346 (17)	H14⋯C21^iv^	2.899 (18)
O1⋯H9	2.406 (16)	H14⋯C22^iv^	2.990 (18)
O1⋯H16*B*	2.345 (16)	H15⋯C19^iv^	2.951 (18)
O1⋯H4^vi^	2.51 (2)	H16*B*⋯S1^v^	2.852 (16)
N1⋯S1	3.0801 (11)	H16*B*⋯C1^v^	2.973 (16)
N1⋯H22	2.552 (15)	H18⋯C6^v^	2.934 (19)
C1⋯C12^v^	3.4639 (18)	H5⋯H16*A*	2.16 (2)
C1⋯C13^v^	3.4372 (18)	H12⋯H21^i^	2.46 (3)
C2⋯C12^v^	3.541 (2)	H15⋯H21^viii^	2.51 (3)
C3⋯C3^vii^	3.485 (2)	H16*B*⋯H18	2.51 (2)
C5⋯C22	3.4988 (19)	H18⋯H22^v^	2.53 (2)
C5⋯C17	3.4201 (18)		

Symmetry codes: (i) 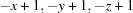; (ii) 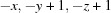; (iii) 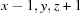; (iv) 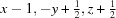; (v) 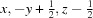; (vi) 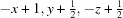; (vii) 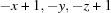; (viii) 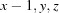.

**Table 3:** Comparison of the selected (X-ray and DFT) geometric data (Å, °)

Bonds/angles	X-ray	B3LYP/6–311G(d,p)
Cl1—C11	1.7357 (13)	1.80981
Cl2—C13	1.7382 (13)	1.80489
S1—C8	1.7525 (12)	1.80120
S1—C1	1.7561 (13)	1.82629
O1—C7	1.2228 (16)	1.23968
N1—C7	1.3759 (16)	1.38157
N1—C6	1.4192 (16)	1.41776
N1—C16	1.4661 (16)	1.47048
C8—S1—C1	100.14 (6)	98.69028
C7—N1—C6	125.51 (10)	124.58623
C7—N1—C16	115.14 (10)	116.12685
C6—N1—C16	119.20 (10)	119.26679
C2—C1—C6	120.71 (12)	121.24260
C2—C1—S1	117.26 (10)	117.48822
C6—C1—S1	122.02 (10)	121.26667

**Table 4:** Calculated energies.

Mol­ecular Energy (a.u.) (eV)	Compound (I)
Total Energy *TE* (eV)	−62249, 6662
*E*_HOMO_ (eV)	−8.2479
*E*_LUMO_ (eV)	−2.9115
Gap Δ*E* (eV)	5.3364
Dipole moment, μ (Debye)	3.4723
Ionization potential, *I* (eV)	8.2479
Electron affinity, *A*	2.9115
Electro negativity, χ	5.3364
Hardness, η	2.6682
Electrophilicity index, ω	5.8340
Softness, σ	0.3748
Fraction of electron transferred, Δ*N*	0.2662

**Table 5:** Experimental details

Crystal data	
Chemical formula	C_22_H_15_Cl_2_NOS
*M*_r_	412.31
Crystal system, space group	Monoclinic, *P*2_1_/*c*
Temperature (K)	150
*a*, *b*, *c* (Å)	9.0373 (7), 16.6798 (13), 12.511 (1)
β (°)	95.982 (2)
*V* (Å^3^)	1875.6 (3)
*Z*	4
Radiation type	Cu *K*α
μ (mm^−1^)	4.25
Crystal size (mm)	0.15 × 0.13 × 0.09

Data collection	
Diffractometer	Bruker D8 VENTURE PHOTON 100 CMOS
Absorption correction	Numerical (*SADABS*; Krause *et al.*, 2015 ▸)
*T*_min_, *T*_max_	0.59, 0.70
No. of measured, independent and observed [*I* > 2σ(*I*)] reflections	48886, 3847, 3650
*R*_int_	0.038
(sin θ/λ)_max_ (Å^−1^)	0.625

Refinement	
*R*[*F* ^2^ > 2σ(*F* ^2^)], *wR*(*F* ^2^), *S*	0.026, 0.070, 1.05
No. of reflections	3847
No. of parameters	304
H-atom treatment	All H-atom parameters refined
Δρ_max_, Δρ_min_ (e Å^−3^)	0.22, −0.26

Computer programs: *APEX3* (Bruker, 2016 ▸), *SAINT* (Bruker, 2016 ▸), *SHELXT* (Sheldrick, 2015*a* ▸), *SHELXL2018* (Sheldrick, 2015*b* ▸), *DIAMOND* (Brandenburg & Putz, 2012 ▸) and *SHELXTL* (Bruker, 2016 ▸).

## supplementary crystallographic information

### Crystal data

**Table:**

C_22_H_15_Cl_2_NOS	*F*(000) = 848
*M**_r_* = 412.31	*D*_x_ = 1.460 Mg m^−^^3^
Monoclinic, *P*2_1_/*c*	Cu *K*α radiation, λ = 1.54178 Å
*a* = 9.0373 (7) Å	Cell parameters from 9943 reflections
*b* = 16.6798 (13) Å	θ = 4.4–43.5°
*c* = 12.511 (1) Å	µ = 4.25 mm^−^^1^
β = 95.982 (2)°	*T* = 150 K
*V* = 1875.6 (3) Å^3^	Block, colourless
*Z* = 4	0.14 × 0.13 × 0.09 mm

### Data collection

**Table:**

Bruker D8 VENTURE PHOTON 100 CMOS diffractometer	3847 independent reflections
Radiation source: INCOATEC IµS micro-focus source	3650 reflections with *I* > 2σ(*I*)
Mirror monochromator	*R*_int_ = 0.038
Detector resolution: 10.4167 pixels mm^-1^	θ_max_ = 74.6°, θ_min_ = 4.4°
ω scans	*h* = −11→11
Absorption correction: numerical (*SADABS*; Krause *et al.*, 2015)	*k* = −20→20
*T*_min_ = 0.59, *T*_max_ = 0.70	*l* = −15→15
48886 measured reflections	

### Refinement

**Table:**

Refinement on *F*^2^	Secondary atom site location: difference Fourier map
Least-squares matrix: full	Hydrogen site location: difference Fourier map
*R*[*F*^2^ > 2σ(*F*^2^)] = 0.026	All H-atom parameters refined
*wR*(*F*^2^) = 0.070	*w* = 1/[σ^2^(*F*_o_^2^) + (0.0379*P*)^2^ + 0.6937*P*] where *P* = (*F*_o_^2^ + 2*F*_c_^2^)/3
*S* = 1.05	(Δ/σ)_max_ = 0.001
3847 reflections	Δρ_max_ = 0.22 e Å^−^^3^
304 parameters	Δρ_min_ = −0.26 e Å^−^^3^
0 restraints	

### Special details

**Table:**

Geometry. All esds (except the esd in the dihedral angle between two l.s. planes) are estimated using the full covariance matrix. The cell esds are taken into account individually in the estimation of esds in distances, angles and torsion angles; correlations between esds in cell parameters are only used when they are defined by crystal symmetry. An approximate (isotropic) treatment of cell esds is used for estimating esds involving l.s. planes.
Refinement. Refinement of F^2^ against ALL reflections. The weighted R-factor wR and goodness of fit S are based on F^2^, conventional R-factors R are based on F, with F set to zero for negative F^2^. The threshold expression of F^2^ > 2sigma(F^2^) is used only for calculating R-factors(gt) etc. and is not relevant to the choice of reflections for refinement. R-factors based on F^2^ are statistically about twice as large as those based on F, and R- factors based on ALL data will be even larger.

### Fractional atomic coordinates and isotropic or equivalent isotropic displacement parameters (Å^2^)

**Table:**

	*x*	*y*	*z*	*U*_iso_*/*U*_eq_	
Cl1	0.32725 (3)	0.51603 (2)	0.51778 (3)	0.03318 (9)	
Cl2	−0.08376 (4)	0.45362 (2)	0.78449 (3)	0.03443 (10)	
S1	0.15102 (3)	0.21109 (2)	0.37534 (2)	0.02451 (9)	
O1	0.29675 (11)	0.36735 (6)	0.18264 (8)	0.0318 (2)	
N1	0.36671 (11)	0.23777 (6)	0.20360 (8)	0.0231 (2)	
C1	0.29233 (13)	0.14621 (7)	0.34225 (10)	0.0235 (2)	
C2	0.30706 (15)	0.07308 (8)	0.39669 (11)	0.0287 (3)	
H2	0.2413 (19)	0.0621 (10)	0.4526 (14)	0.033 (4)*	
C3	0.41257 (16)	0.01777 (8)	0.37140 (12)	0.0327 (3)	
H3	0.4187 (18)	−0.0323 (10)	0.4058 (13)	0.030 (4)*	
C4	0.50780 (16)	0.03719 (8)	0.29531 (13)	0.0330 (3)	
H4	0.579 (2)	−0.0001 (12)	0.2780 (15)	0.042 (5)*	
C5	0.49570 (15)	0.11045 (8)	0.24245 (11)	0.0285 (3)	
H5	0.5655 (19)	0.1230 (11)	0.1921 (13)	0.035 (4)*	
C6	0.38507 (13)	0.16533 (7)	0.26301 (10)	0.0233 (2)	
C7	0.29726 (13)	0.30575 (7)	0.23567 (10)	0.0237 (2)	
C8	0.22305 (13)	0.30312 (7)	0.33731 (10)	0.0223 (2)	
C9	0.20587 (14)	0.37330 (7)	0.38765 (10)	0.0241 (2)	
H9	0.2472 (18)	0.4183 (10)	0.3553 (13)	0.031 (4)*	
C10	0.13615 (13)	0.38965 (7)	0.48567 (10)	0.0232 (2)	
C11	0.18203 (13)	0.45583 (7)	0.55067 (10)	0.0239 (2)	
C12	0.11763 (15)	0.47512 (8)	0.64272 (11)	0.0266 (3)	
H12	0.152 (2)	0.5194 (11)	0.6845 (14)	0.040 (5)*	
C13	0.00202 (14)	0.42783 (8)	0.67128 (10)	0.0261 (3)	
C14	−0.04785 (15)	0.36229 (8)	0.61082 (11)	0.0283 (3)	
H14	−0.130 (2)	0.3302 (11)	0.6306 (14)	0.038 (4)*	
C15	0.01925 (15)	0.34384 (8)	0.51908 (11)	0.0271 (3)	
H15	−0.019 (2)	0.3021 (11)	0.4758 (15)	0.042 (5)*	
C16	0.43514 (15)	0.24544 (8)	0.10287 (10)	0.0261 (3)	
H16A	0.4342 (18)	0.1915 (10)	0.0694 (13)	0.029 (4)*	
H16B	0.3717 (18)	0.2771 (10)	0.0550 (13)	0.027 (4)*	
C17	0.59004 (14)	0.28115 (7)	0.11411 (10)	0.0235 (2)	
C18	0.65243 (16)	0.30239 (9)	0.02058 (11)	0.0311 (3)	
H18	0.593 (2)	0.2943 (11)	−0.0487 (15)	0.039 (5)*	
C19	0.79411 (17)	0.33523 (9)	0.02566 (13)	0.0384 (3)	
H19	0.835 (2)	0.3503 (12)	−0.0383 (16)	0.050 (5)*	
C20	0.87604 (17)	0.34739 (9)	0.12401 (14)	0.0382 (3)	
H20	0.973 (2)	0.3695 (12)	0.1271 (15)	0.046 (5)*	
C21	0.81497 (16)	0.32701 (8)	0.21707 (13)	0.0334 (3)	
H21	0.874 (2)	0.3354 (11)	0.2875 (14)	0.040 (5)*	
C22	0.67257 (15)	0.29448 (8)	0.21258 (11)	0.0273 (3)	
H22	0.6283 (17)	0.2799 (9)	0.2793 (12)	0.024 (4)*	

### Atomic displacement parameters (Å^2^)

**Table:**

	*U*^11^	*U*^22^	*U*^33^	*U*^12^	*U*^13^	*U*^23^
Cl1	0.02604 (16)	0.03290 (17)	0.04160 (19)	−0.00735 (12)	0.00829 (13)	−0.00211 (13)
Cl2	0.03839 (18)	0.03495 (18)	0.03231 (17)	0.00967 (13)	0.01489 (13)	0.00164 (12)
S1	0.02266 (15)	0.02109 (15)	0.03075 (17)	−0.00207 (11)	0.00738 (12)	0.00098 (11)
O1	0.0405 (5)	0.0264 (5)	0.0303 (5)	0.0018 (4)	0.0116 (4)	0.0066 (4)
N1	0.0226 (5)	0.0248 (5)	0.0224 (5)	−0.0006 (4)	0.0050 (4)	0.0000 (4)
C1	0.0225 (6)	0.0217 (6)	0.0261 (6)	−0.0018 (5)	0.0009 (5)	−0.0017 (5)
C2	0.0297 (7)	0.0241 (6)	0.0321 (7)	−0.0029 (5)	0.0017 (5)	0.0022 (5)
C3	0.0349 (7)	0.0215 (6)	0.0406 (8)	0.0005 (5)	−0.0015 (6)	0.0025 (5)
C4	0.0295 (7)	0.0245 (6)	0.0446 (8)	0.0037 (5)	0.0022 (6)	−0.0047 (6)
C5	0.0253 (6)	0.0267 (6)	0.0337 (7)	−0.0007 (5)	0.0044 (5)	−0.0047 (5)
C6	0.0225 (6)	0.0214 (6)	0.0255 (6)	−0.0026 (5)	0.0004 (5)	−0.0020 (5)
C7	0.0223 (6)	0.0241 (6)	0.0247 (6)	−0.0015 (5)	0.0025 (5)	0.0010 (5)
C8	0.0194 (5)	0.0231 (6)	0.0246 (6)	0.0002 (4)	0.0034 (4)	0.0033 (4)
C9	0.0229 (6)	0.0224 (6)	0.0276 (6)	0.0000 (5)	0.0051 (5)	0.0036 (5)
C10	0.0228 (6)	0.0207 (6)	0.0265 (6)	0.0038 (5)	0.0041 (5)	0.0034 (4)
C11	0.0201 (6)	0.0224 (6)	0.0293 (6)	0.0025 (4)	0.0034 (5)	0.0033 (5)
C12	0.0259 (6)	0.0242 (6)	0.0293 (6)	0.0039 (5)	0.0015 (5)	−0.0006 (5)
C13	0.0265 (6)	0.0260 (6)	0.0267 (6)	0.0083 (5)	0.0069 (5)	0.0042 (5)
C14	0.0279 (6)	0.0238 (6)	0.0350 (7)	0.0014 (5)	0.0111 (5)	0.0048 (5)
C15	0.0281 (6)	0.0222 (6)	0.0318 (7)	−0.0001 (5)	0.0075 (5)	0.0002 (5)
C16	0.0273 (6)	0.0315 (7)	0.0196 (6)	−0.0010 (5)	0.0033 (5)	−0.0020 (5)
C17	0.0256 (6)	0.0215 (6)	0.0240 (6)	0.0032 (5)	0.0062 (5)	0.0002 (4)
C18	0.0344 (7)	0.0333 (7)	0.0271 (7)	0.0065 (6)	0.0104 (5)	0.0039 (5)
C19	0.0385 (8)	0.0330 (7)	0.0476 (9)	0.0061 (6)	0.0232 (7)	0.0101 (6)
C20	0.0270 (7)	0.0262 (7)	0.0630 (10)	−0.0002 (5)	0.0123 (6)	0.0026 (6)
C21	0.0290 (7)	0.0262 (7)	0.0442 (8)	−0.0002 (5)	−0.0002 (6)	−0.0042 (6)
C22	0.0294 (6)	0.0261 (6)	0.0265 (6)	−0.0009 (5)	0.0042 (5)	−0.0013 (5)

### Geometric parameters (Å, º)

**Table:**

Cl1—C11	1.7357 (13)	C10—C11	1.4076 (18)
Cl2—C13	1.7382 (13)	C11—C12	1.3814 (18)
S1—C8	1.7525 (12)	C12—C13	1.3854 (19)
S1—C1	1.7561 (13)	C12—H12	0.940 (19)
O1—C7	1.2228 (16)	C13—C14	1.3781 (19)
N1—C7	1.3759 (16)	C14—C15	1.3874 (19)
N1—C6	1.4192 (16)	C14—H14	0.971 (19)
N1—C16	1.4661 (16)	C15—H15	0.928 (19)
C1—C2	1.3967 (18)	C16—C17	1.5143 (18)
C1—C6	1.4000 (18)	C16—H16A	0.993 (17)
C2—C3	1.387 (2)	C16—H16B	0.945 (16)
C2—H2	0.981 (18)	C17—C22	1.3899 (18)
C3—C4	1.387 (2)	C17—C18	1.3965 (18)
C3—H3	0.938 (17)	C18—C19	1.388 (2)
C4—C5	1.388 (2)	C18—H18	0.979 (18)
C4—H4	0.94 (2)	C19—C20	1.383 (2)
C5—C6	1.3990 (18)	C19—H19	0.95 (2)
C5—H5	0.960 (18)	C20—C21	1.382 (2)
C7—C8	1.4988 (17)	C20—H20	0.95 (2)
C8—C9	1.3458 (18)	C21—C22	1.392 (2)
C9—C10	1.4616 (17)	C21—H21	0.993 (18)
C9—H9	0.948 (17)	C22—H22	0.992 (16)
C10—C15	1.4024 (18)		
			
Cl1···Cl1^i^	3.2439 (5)	C6···C22	3.4830 (18)
Cl1···C14^ii^	3.4981 (14)	C6···C12^v^	3.5828 (18)
Cl1···H9	2.647 (16)	C7···C22	3.4391 (18)
Cl2···H19^iii^	2.96 (2)	C10···C12^ii^	3.4871 (18)
Cl2···H9^ii^	3.044 (16)	C14···C20^iv^	3.572 (2)
Cl2···H4^iv^	3.138 (18)	C5···H16A	2.563 (16)
S1···Cl2^v^	3.5832 (5)	C6···H22	2.904 (15)
S1···Cl2^v^	3.5832 (5)	C8···H15	2.929 (18)
S1···N1	3.0801 (11)	C16···H5	2.556 (18)
S1···C15	3.1625 (14)	C17···H5	2.829 (18)
S1···C13^v^	3.6033 (13)	C18···H3^vi^	2.998 (17)
S1···H15	2.578 (18)	C21···H12^i^	2.845 (18)
O1···C17	3.2096 (16)	H14···C20^iv^	2.964 (18)
O1···C4^vi^	3.3346 (17)	H14···C21^iv^	2.899 (18)
O1···H9	2.406 (16)	H14···C22^iv^	2.990 (18)
O1···H16B	2.345 (16)	H15···C19^iv^	2.951 (18)
O1···H4^vi^	2.51 (2)	H16B···S1^v^	2.852 (16)
N1···S1	3.0801 (11)	H16B···C1^v^	2.973 (16)
N1···H22	2.552 (15)	H18···C6^v^	2.934 (19)
C1···C12^v^	3.4639 (18)	H5···H16A	2.16 (2)
C1···C13^v^	3.4372 (18)	H12···H21^i^	2.46 (3)
C2···C12^v^	3.541 (2)	H15···H21^viii^	2.51 (3)
C3···C3^vii^	3.485 (2)	H16B···H18	2.51 (2)
C5···C22	3.4988 (19)	H18···H22^v^	2.53 (2)
C5···C17	3.4201 (18)		
			
C8—S1—C1	100.14 (6)	C11—C12—C13	118.49 (12)
C7—N1—C6	125.51 (10)	C11—C12—H12	120.1 (11)
C7—N1—C16	115.14 (10)	C13—C12—H12	121.4 (11)
C6—N1—C16	119.20 (10)	C14—C13—C12	121.49 (12)
C2—C1—C6	120.71 (12)	C14—C13—Cl2	119.70 (10)
C2—C1—S1	117.26 (10)	C12—C13—Cl2	118.79 (10)
C6—C1—S1	122.02 (10)	C13—C14—C15	118.94 (12)
C3—C2—C1	120.17 (13)	C13—C14—H14	120.9 (10)
C3—C2—H2	121.4 (10)	C15—C14—H14	120.1 (10)
C1—C2—H2	118.5 (10)	C14—C15—C10	122.24 (12)
C4—C3—C2	119.47 (13)	C14—C15—H15	118.7 (12)
C4—C3—H3	120.7 (10)	C10—C15—H15	118.9 (12)
C2—C3—H3	119.8 (10)	N1—C16—C17	115.04 (10)
C3—C4—C5	120.59 (13)	N1—C16—H16A	107.3 (9)
C3—C4—H4	119.6 (12)	C17—C16—H16A	111.3 (9)
C5—C4—H4	119.7 (12)	N1—C16—H16B	108.1 (10)
C4—C5—C6	120.71 (13)	C17—C16—H16B	109.4 (10)
C4—C5—H5	118.7 (10)	H16A—C16—H16B	105.2 (13)
C6—C5—H5	120.6 (11)	C22—C17—C18	118.42 (12)
C5—C6—C1	118.24 (12)	C22—C17—C16	123.41 (11)
C5—C6—N1	120.50 (11)	C18—C17—C16	118.16 (12)
C1—C6—N1	121.26 (11)	C19—C18—C17	120.85 (14)
O1—C7—N1	120.68 (11)	C19—C18—H18	120.7 (11)
O1—C7—C8	120.54 (11)	C17—C18—H18	118.5 (11)
N1—C7—C8	118.78 (10)	C20—C19—C18	120.29 (14)
C9—C8—C7	117.09 (11)	C20—C19—H19	119.3 (12)
C9—C8—S1	124.79 (10)	C18—C19—H19	120.4 (12)
C7—C8—S1	117.88 (9)	C21—C20—C19	119.32 (14)
C8—C9—C10	129.48 (12)	C21—C20—H20	120.6 (11)
C8—C9—H9	114.8 (10)	C19—C20—H20	120.1 (11)
C10—C9—H9	115.7 (10)	C20—C21—C22	120.69 (14)
C15—C10—C11	116.15 (11)	C20—C21—H21	119.2 (11)
C15—C10—C9	123.53 (12)	C22—C21—H21	120.1 (11)
C11—C10—C9	120.29 (11)	C17—C22—C21	120.43 (13)
C12—C11—C10	122.68 (12)	C17—C22—H22	118.7 (9)
C12—C11—Cl1	117.23 (10)	C21—C22—H22	120.9 (9)
C10—C11—Cl1	120.08 (10)		
			
C8—S1—C1—C2	155.71 (10)	S1—C8—C9—C10	4.6 (2)
C8—S1—C1—C6	−25.73 (11)	C8—C9—C10—C15	−29.8 (2)
C6—C1—C2—C3	−1.12 (19)	C8—C9—C10—C11	152.34 (13)
S1—C1—C2—C3	177.47 (10)	C15—C10—C11—C12	0.42 (18)
C1—C2—C3—C4	3.0 (2)	C9—C10—C11—C12	178.46 (11)
C2—C3—C4—C5	−1.7 (2)	C15—C10—C11—Cl1	179.25 (9)
C3—C4—C5—C6	−1.5 (2)	C9—C10—C11—Cl1	−2.71 (16)
C4—C5—C6—C1	3.35 (19)	C10—C11—C12—C13	−0.78 (19)
C4—C5—C6—N1	−175.72 (12)	Cl1—C11—C12—C13	−179.64 (9)
C2—C1—C6—C5	−2.05 (18)	C11—C12—C13—C14	0.72 (19)
S1—C1—C6—C5	179.43 (9)	C11—C12—C13—Cl2	−177.88 (9)
C2—C1—C6—N1	177.02 (11)	C12—C13—C14—C15	−0.33 (19)
S1—C1—C6—N1	−1.50 (17)	Cl2—C13—C14—C15	178.27 (10)
C7—N1—C6—C5	−158.93 (12)	C13—C14—C15—C10	0.0 (2)
C16—N1—C6—C5	16.29 (17)	C11—C10—C15—C14	0.00 (19)
C7—N1—C6—C1	22.03 (18)	C9—C10—C15—C14	−177.97 (12)
C16—N1—C6—C1	−162.76 (11)	C7—N1—C16—C17	84.01 (14)
C6—N1—C7—O1	174.42 (12)	C6—N1—C16—C17	−91.69 (14)
C16—N1—C7—O1	−0.96 (17)	N1—C16—C17—C22	9.68 (18)
C6—N1—C7—C8	−5.15 (18)	N1—C16—C17—C18	−169.73 (11)
C16—N1—C7—C8	179.46 (10)	C22—C17—C18—C19	0.7 (2)
O1—C7—C8—C9	−23.67 (18)	C16—C17—C18—C19	−179.87 (13)
N1—C7—C8—C9	155.91 (11)	C17—C18—C19—C20	0.1 (2)
O1—C7—C8—S1	150.91 (10)	C18—C19—C20—C21	−0.4 (2)
N1—C7—C8—S1	−29.52 (15)	C19—C20—C21—C22	0.1 (2)
C1—S1—C8—C9	−145.73 (11)	C18—C17—C22—C21	−1.06 (19)
C1—S1—C8—C7	40.15 (10)	C16—C17—C22—C21	179.53 (12)
C7—C8—C9—C10	178.71 (12)	C20—C21—C22—C17	0.7 (2)

Symmetry codes: (i) −*x*+1, −*y*+1, −*z*+1; (ii) −*x*, −*y*+1, −*z*+1; (iii) *x*−1, *y*, *z*+1; (iv) *x*−1, −*y*+1/2, *z*+1/2; (v) *x*, −*y*+1/2, *z*−1/2; (vi) −*x*+1, *y*+1/2, −*z*+1/2; (vii) −*x*+1, −*y*, −*z*+1; (viii) *x*−1, *y*, *z*.

### Hydrogen-bond geometry (Å, º)

**Table:**

*D*—H···*A*	*D*—H	H···*A*	*D*···*A*	*D*—H···*A*
C4—H4···O1^ix^	0.936 (19)	2.51 (2)	3.3346 (17)	147.7 (15)
C16—H16*B*···S1^v^	0.945 (16)	2.852 (16)	3.7011 (13)	149.9 (12)
C3—H3···*Cg*4^ix^	0.938 (17)	2.901 (17)	3.6428 (15)	136.8 (13)
C14—H14···*Cg*4^x^	0.971 (19)	2.710 (18)	3.5593 (15)	146.8 (14)
C18—H18···*Cg*1^xi^	0.979 (18)	2.969 (18)	3.6759 (16)	130.0 (13)

Symmetry codes: (v) *x*, −*y*+1/2, *z*−1/2; (ix) −*x*+1, *y*−1/2, −*z*+1/2; (x) *x*−1, −*y*−1/2, *z*−1/2; (xi) *x*, −*y*−1/2, *z*−3/2.
